# Exercise-induced changes to the fiber type-specific redox state in human skeletal muscle are associated with aerobic capacity

**DOI:** 10.1152/japplphysiol.00662.2022

**Published:** 2023-07-20

**Authors:** James Shadiow, Edwin R. Miranda, Ryan K. Perkins, Corey E. Mazo, Zhen Lin, Kendell N. Lewis, Jacob T. Mey, Thomas P. J. Solomon, Jacob M. Haus

**Affiliations:** ^1^School of Kinesiology, University of Michigan, Ann Arbor, Michigan, United States; ^2^Pennington Biomedical Research Center, Louisiana State University, Baton Rouge, Louisiana, United States; ^3^Blazon Scientific, London, United Kingdom

**Keywords:** autofluorescence, exercise physiology, flavoproteins, NAD/NADH, oxygen delivery

## Abstract

The benefits of exercise involve skeletal muscle redox state alterations of nicotinamide adenine dinucleotide (NAD) and flavin adenine dinucleotide (FAD). We determined the fiber-specific effects of acute exercise on the skeletal muscle redox state in healthy adults. Muscle biopsies were obtained from 19 participants (11 M, 8 F; 26 ± 4 yr) at baseline (fasted) and 30 min and 3 h after treadmill exercise at 80% maximal oxygen consumption (V̇o_2max_). Muscle samples were probed for autofluorescence of NADH (excitation at 340–360 nm) and oxidized flavoproteins (Fp; excitation at 440–470 nm) and subsequently, fiber typed to quantify the redox signatures of individual muscle fibers. Redox state was calculated as the oxidation-to-reduction redox ratio: Fp/(Fp + NADH). At baseline, pair-wise comparisons revealed that the redox ratio of myosin heavy chain (MHC) I fibers was 7.2% higher than MHC IIa (*P* = 0.023, 95% CI: 5.2, 9.2%) and the redox ratio of MHC IIa was 8.0% higher than MHC IIx (*P* = 0.035, 95% CI: 6.8, 9.2%). MHC I fibers also displayed greater NADH intensity than MHC IIx (*P* = 0.007) and greater Fp intensity than both MHC IIa (*P* = 0.019) and MHC IIx (*P* < 0.0001). Fp intensities increased in all fiber types (main effect, *P* = 0.039) but redox ratios did not change (main effect, *P* = 0.483) 30 min after exercise. The change in redox ratio was positively correlated with capillary density in MHC I (rho = 0.762, *P* = 0.037), MHC IIa fibers (rho = 0.881, *P* = 0.007), and modestly in MHC IIx fibers (rho = 0. 771, *P* = 0.103). These findings support the use of redox autofluorescence to interrogate skeletal muscle metabolism.

**NEW & NOTEWORTHY** This study is the first to use autofluorescent imaging to describe differential redox states within human skeletal muscle fiber types with exercise. Our findings highlight an easy and efficacious technique for assessing skeletal muscle redox in humans.

## Graphical abstract

**Figure:**
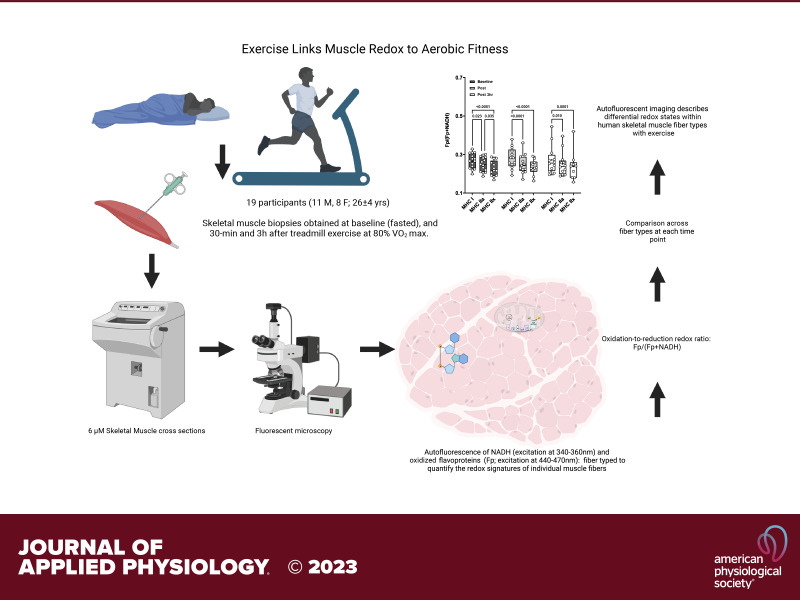

## INTRODUCTION

The redox state, the balance between reduction and oxidation in biochemical processes, regulates energy metabolism, gene expression, posttranslational modification, oxidative stress, and cell death (1, 2). Redox balance is characterized by changes in the levels of the oxidized (FAD and NAD^+^) and reduced (FADH_2_ and NADH) forms of the coenzymes flavin adenine dinucleotide (FAD) and nicotinamide adenine dinucleotide (NAD). These coenzymes are primarily found in the mitochondria where they are involved in oxidative phosphorylation and modulate the interplay between energy metabolism and adaptative gene regulation (1, 3). NAD^+^ is also an important cofactor for cytosolic metabolism and an important cofactor for glycolytic enzymes including GAPDH, LDH, and PDH. Furthermore, NAD+-consuming enzymes such as sirtuins and poly (ADP-ribose) polymerases (PARPs) play a role in the epigenetic regulation of transcription, posttranslational regulation of other cellular enzymes, and DNA damage repair. Therefore, redox biology bridges cellular metabolism with enzyme activities and cellular function.

NAD^+^ availability and/or NAD^+^/NADH ratios decrease with age and are observed in several age-related diseases (3, 4). In rodents, pharmacologically or genetically increasing skeletal muscle NAD^+^ availability and/or NAD^+^/NADH ratios can improve mitochondrial respiratory capacity (5), muscle function (6–8), aging-associated disorders (5, 8), and whole body metabolic health (9). Specifically, inhibition of nicotinamide phosphoribosyltransferase (NAMPT) activity, the rate-limiting enzyme in the NAD salvage pathway, in cultured myotubes resulted in depletion of NAD^+^ consequently inhibiting GAPDH and impairing mitochondrial respiration (10–12). Although studies are limited and largely unconfirmed in humans, individuals with obesity who underwent a 3-wk aerobic exercise training protocol rescued NAMPT protein expression (13), and studies using resistance exercise training in populations of middle-aged, overweight adults found similar increases in skeletal muscle NAMPT expression (14, 15). Indeed, women with obesity significantly improved their glucose disposal rate and improved skeletal muscle insulin signaling shown by increased p-AKT and p-mTOR during a hyperinsulinemic-euglycemic clamp after administration of 250 mg/day for 10 wk of nicotinamide mononucleotide, a main precursor of NAD^+^ (16). Therefore, exercise-induced improvements in mitochondrial respiratory capacity and metabolic health may involve an increase in the skeletal muscle redox ratio. An exercise bout certainly increases energy expenditure and oxidative substrate flux in skeletal muscle, acutely changing the redox state (17, 18). However, skeletal muscle consists of distinct muscle fiber types each with different metabolic and oxidative capacities and mitochondrial contents and activities. Furthermore, skeletal muscle energy metabolism and metabolic flux are also influenced by substrate and oxygen delivery (17, 19). But little is known about fiber type-specific redox signatures and fiber type-specific changes in redox balance during exercise, or whether such changes are related to substrate supply to the muscle. Filling these gaps will bolster mechanistic knowledge of the intracellular changes that occur during exercise.

Measuring muscle redox status can be difficult biochemically due to the labile nature of FAD and NAD between their oxidized (FAD and NAD+) or reduced (FADH_2_ and NADH) states. Fortunately, FAD contained in flavoproteins (Fp) and NADH has unique and measurable autofluorescent signals. Chance et al. (20–25) leveraged these autofluorescent properties in skeletal muscle to derive a sensitive spectrophotometric technique, enabling the study of mitochondrial respiration and the redox ratio between oxidized FAD-containing flavoproteins (Fp) and the sum of oxidized Fp and reduced NAD: Fp/(Fp + NADH). This work was recently advanced by Xu et al. (26, 27) who differentially compared these redox indices within preclinical models of snap-frozen tissues. Their results indicated that autofluorescent methodologies are sensitive enough to detect differences in skeletal muscle redox indices (i.e., Fp, NADH) between young, old, and transgenic muscle-specific NAMPT overexpression in mice. To better understand the responsiveness of muscle to acute exercise stress, we applied these autofluorescence methods to human skeletal muscle and assessed fiber type-specific redox signatures and exercise-induced changes in fiber type-specific redox balance. We also explored the relation between fiber type-specific redox states and characteristics associated with tissue substrate and oxygen delivery, including skeletal muscle capillarization, mitochondrial content, and maximal oxygen consumption (V̇o_2max_). We hypothesized that oxidative fibers would display redox characteristics consistent with greater mitochondrial content and that the autofluorescent-derived metrics would be associated with cardiorespiratory fitness and skeletal muscle capillarization.

## METHODS

### Study Design and Participants

In this current analysis, we included 19 adults (11 M, 8 F) and their baseline characteristics are presented in Table 1. Data from 15 of these individuals have been previously reported (22). This is the first report of these outcomes for the purposes of this manuscript. Inclusion criteria included 18–35 yr of age and free of chronic disease. Exclusion criteria included smoking within the past year, diagnosis of diabetes, cardiovascular disease, kidney disease, major depression, or hypercholesteremia (≥240 mg/dL). All experimental protocols were approved by the Institutional Review Boards of the University of Illinois at Chicago (IRB Approval No.: 2015-0127) and University of Michigan (IRB Approval No.: HUM00174057) due to laboratory relocation. Upon providing verbal and written informed consent, the participants were enrolled and height, weight, body composition via dual X-ray absorptiometry, and maximal aerobic capacity (V̇o_2max_) were collected. All participants displayed moderate levels of physical fitness and were characterized as recreationally active, as defined by the American College of Sports Medicine. To avoid the transient effects of prior maximal exercise, participants returned after approximately 2 wk to perform an acute aerobic exercise at 80% of V̇o_2max_ for 30 min during which muscle biopsies and venous blood were collected from participants before (baseline), 30 min after (post), and 3 h after treadmill exercise. Participants were fasted during the entirety of the visit. Venous blood was used to measure fasting plasma glucose (glucose oxidase reaction; YSI STAT 2300, YSI Life Sciences, Yellow Springs, OH; or Bayer Contour Next Link glucometer, Bayer, Whippany, NJ) and fasting plasma insulin (ELISA 90095, Crystal Chem, Elk Grove Village, IL). These measures were used to calculate homeostatic assessment of insulin resistance (HOMA-IR) (28).

**Table 1.** Participant characteristics

*n*	19 (8 F; 11 M)
Age, yr	26 (24–28)
SBP, mmHg	113 (108–119)
DBP, mmHg	65 (63–68)
BMI, kg/m^2^	24.2 (21.8–26.6)
Body fat, %	25.8 (21.6–30.1)
Lean mass, %	73.2 (69.2–77.2)
Glucose, mg/dL	92 (88–96)
Insulin, mU/L	5.5 (4.4–6.6)
HOMA-IR, A.U.	1.3 (1.0–1.5)
V̇o_2max_, mL/kg/min	46.7 (42.7–50.6)
V̇o_2max_, L/min	3.4 (3.0–3.9)

Data presented as means (95% CI). A.U., arbitrary units; F, female; HOMA-IR, homeostatic model assessment of insulin resistance; M, male; V̇o_2max_, maximal oxygen consumption.

Three days before each visit, participants completed diet and physical activity logs and were asked to replicate them in the days leading up to their subsequent visits. Participants were instructed to abstain from vigorous exercise 48 h before each visit while maintaining their usual physical activity habits. Three-day dietary records were analyzed by retrospective input using the Automated Self-Administered 24-h (ASA24) Dietary Assessment Tool, version (2022), developed by the National Cancer Institute, Bethesda, MD (29). Goldberg cutoffs (Energy Intake: Basal Metabolic Rate; EI:BMR) (30) with Black adjustments (31) using cohort-derived viability in daily energy intake reporting were used to assess dietary records for implausible under- or overreporting of dietary intake. Estimated BMR was calculated from established regression models using age, sex, fat mass, and fat-free mass (32). Physical activity level was estimated from V̇o_2max_ according to ACSM criteria (33) and historical physical activity coefficients were applied to BMR to estimate total daily energy expenditure (34). Plausible reporting as a cohort was set using the calculated Goldberg cutoffs of an EI:BMR between 1.18 and 1.55. Plausible reporting for individuals was set using the calculated Goldberg cutoffs of an EI:BMR between 0.76 and 2.41. Dietary intake was strikingly similar at baseline and end of study visits (Supplemental Table S1; all Supplemental material is available at https://doi.org/10.6084/m9.figshare.23564385). Dietary intake was consistent in individuals between study visits (Supplemental Table S1). Dietary reporting was plausible and outperformed plausible reporting accuracy compared with other US cohorts (35). Individual-level dietary records included three total occurrences of plausible underreporting (baseline: 2, end of study: 1).

Participants were also instructed to abstain from vigorous exercise and alcohol consumption 48 h before each visit, and caffeine consumption 24 h before each visit. Participants were asked to arrive at each visit by sedentary means (i.e., car, public transportation, etc.) having fasted for at least 12 h. Testing of all participants was done between 0700 and 0900 to account for diurnal variations in outcome measures and to minimize the participants’ burden from being fasted.

### V̇o_2max_ and Acute Exercise Bout

V̇o_2max_ was determined using a treadmill ramp protocol during which the participants ran at a self-selected speed while the treadmill grade increased 2% every 2-min until volitional fatigue was reached. Participants self-selected their speed to reach their max after 8–12 min. Expired air was collected for the duration of the test and was analyzed via the PARVO Medics metabolic cart (Salt Lake City, UT). Heart rate was monitored continuously via Polar H10 heart rate monitors (Polar Electro Inc., Kempele, Finland), and ratings of perceived exertion (RPE; Borg scale 6–20) were assessed every 2 min. As described previously (36) and according to criteria set by the American College of Sports Medicine Guidelines for Exercise Testing and Prescription (37), V̇o_2max_ was achieved if the participants met three of the four criteria: a plateau in oxygen consumption (V̇o_2_) defined as an increase in two or more consecutive 1-min mean V̇o_2_ values of less than 1.5 mL/min/kg despite an increase in workload, an RPE >17, RER >1.1, and a HR >85% age-predicted maximal heart rate. Within 2 wk, participants completed an acute exercise bout on the treadmill at 80% V̇o_2max_ for 30 min. Expired air was monitored as described earlier to confirm target V̇o_2_ and collect expired gases. Indirect calorimetry outcomes of the acute exercise bout are presented in Table 2. To achieve target V̇o_2_, speed/incline adjustments were made throughout the bout.

**Table 2.** Indirect calorimetry during exercise at 80% V̇o_2max_

RER, AU	0.92 (0.90–0.93)
FAT, %	28.1 (23.7–32.5)
CHO, %	71.9 (67.5–76.2)
FAT energy expended, kcal	107.7 (88.8–126.5)
CHO energy expended, kcal	277.0 (235.8–318.2)

Data presented as means (95% CI). CHO, carbohydrates; RER, respiratory exchange ratio; V̇o_2max_, maximal oxygen consumption.

### Skeletal Muscle Biopsy

Skeletal muscle biopsies were taken at the final study visit from the vastus lateralis before exercise (baseline), 30 min postexercise (post), and 3 h postexercise (post 3 h) as previously described (36). Briefly, after 30 min of supine rest, 2% lidocaine HCl without epinephrine was locally administered subcutaneously. A small incision (∼0.5 cm) was made and ∼200 mg of muscle tissue was extracted using a Bergström needle with suction. Muscle tissue was cleared of all visible connective tissue and fat, blotted with gauze to remove blood, embedded in optimal cutting temperature compound (OCT), and then immediately frozen in liquid nitrogen-cooled isopentane, and stored at −80°C for future analysis.

### Redox Autofluorescence

Redox analysis of unfixed frozen muscle sections via autofluorescence was adapted from methods previously described and the redox state was calculated as Fp/(Fp + NADH) (26, 27). Sections (6 μm) were generated via microtome-cryostat (Microm HM 525, Microm International GmbH, Walldorf, Germany) at −20°C and immediately imaged for autofluorescence. All images were captured using the BZ-X710 all-in-one fluorescence microscope (Keyence, Itasca, IL) at ×20 magnification. NADH autofluorescence was captured under DAPI filter (excitation: 340–360 nm; emission: 450–460 nm) and Fp autofluorescence was captured under GFP filter (excitation: 440–470 nm; emission: 525–550 nm). Autofluorescence was internally validated using serial dilutions of β-NADH (Sigma-Aldrich; St. Louis, MO; Cat. No.: N1161; dissolved in 0.01 M NaOH) encompassing the physiological range from 1 mM to 0.01 mM (38) in a 96-well plate resulting in R^2^ = 0.999 (see Supplemental Fig. S1). Each dilution was run in triplicate. The average interassay variability was 8.9%.

### Immunofluorescence

Fiber type staining and analysis were adapted from methods previously described (39). Sections of 6 μm were fixed with 1:1 acetone/methanol solution for 10 min at room temperature (RT), washed with PBS supplemented with 0.01% Tween-20 (PBST) three times for 5 min, and blocked for 30 min with 5% donkey serum in PBS then washed with PBST again three times for 5 min. Sections were then incubated with myosin heavy chain (MHC) IIa antibody (1:80, Developmental Studies Hybridoma Bank; Iowa City, IA; Cat. No.: A4.74) for 30 min at 37°C, washed, and incubated with secondary antibody (1:500, Abcam; Cambridge, UK; Cat. No.: ab150106) for 30 min at 37°C. Following MHC IIa antibody incubation, sections were blocked with 5% donkey serum in PBS and then washed. Sections were then incubated with MHC I antibody (1:100, Developmental Studies Hybridoma Bank; Cat. No.: BA-D5) for 30 min at 37°C, washed, then incubated with secondary antibody (1:500, Abcam; Cat. No.: ab150107) for 30 min at 37°C, washed, stained with DAPI (1:10,000, Thermo; Waltham, MA; Cat. No.: 62248) for 3 min, then washed again. MHC isoform specificities of these antibodies were previously confirmed by Western blot (39).

For mitochondrial markers, a single 6-μm section was fixed and blocked as described in *Redox Autofluorescence*. Sections were incubated overnight at 4°C with either succinate dehydrogenase subunit A (SDHA) (1:125, Abcam; Cat. No.: ab14715) or citrate synthase (CS) (1:100, Santa Cruz Biotechnology, Inc.; Dallas, TX; Cat. No.: sc-390693). Antibodies were internally validated for specificity against isotype and secondary only control treated slides. Sections were then washed, and incubated with secondary antibody (1:500, Abcam; Cat. No.: ab150107) for 30 min at RT, washed, then incubated with DAPI (1:10,000, Thermo Fisher, Waltham, MA, Cat. No.: 62248), and then washed again. All primary and secondary antibodies were diluted in 1% donkey serum in PBS. All slides were mounted with ProLong Gold mounting medium (Thermo Fisher, Cat. No.: MP36930) with coverslip before imaging.

### Image Analysis

Discernable MHC I, MHC IIa, and MHC IIx fibers within the ×20 field of view were traced and quantified on the accompanied autofluorescence and mitochondrial content-stained image using BZ-X Analysis Software (Keyence). Myofibers positive for MHC I and negative for MHC IIa were classified as type I, fibers positive for MHC IIa and negative for MHC I were classified as type IIa, and fibers negative for both MHC I and MHC IIa were classified as type IIx. Myofibers coexpressing more than one MHC isoform were classified as hybrid fibers and excluded from analyses. The number of myofibers included in analyses were 65 ± 10. Nuclei density was assessed by computational counts of DAPI-stained nuclei. Muscle fiber type and nuclei characteristics are presented in Table 3. For measurable comparisons, fiber-specific NADH and Fp signals were standardized to the same exposure time, instrument gain, and light source intensity. Exposure time was optimized so that cytosolic compartments were free of saturation. Background signal intensity was subtracted from all fluorescence imaging signal intensities when calculating relative intensities specific to each fiber type.

**Table 3.** Vastus lateralis muscle characteristics

Fiber-type distribution, %	
MHC I	45.9 (37.0–54.8)
MHC IIa	44.5 (35.8–53.1)
MHC IIx	9.6 (4.9–14.3)
Fiber-type area, μm^2^	
MHC I	5974.3 (4676.3–7272.2)
MHC IIa	5867.0 (5112.3–6621.6)
MHC IIx	4109.9 (3374.3–4845.5)
Capillarization	
Capillary density, mm^2^	398.4 (359.1–437.7)
C:F, AU	2.3 (2.0–2.5)
CC, AU	3.6 (3.2–4.0)
CC/FA, μm^2^·1,000	0.7 (0.6–0.8)
Sharing factor, AU	1.7 (1.5–1.9)
Max DD, μm	34.9 (32.4–37.4)
Avg DD, μm	18.3 (17.1–20.0)
Nuclei density, mm^2^	781.5 (662.4–900.7)

Data presented as means (95% CI). Avg DD, estimated maximal O_2_ diffusion distance; C:F, capillary-to-fiber ratio; CC, average number of capillary contacts per fiber; CC/FA, average number of capillary contacts per fiber area (FA); max DD, estimated maximal O_2_ diffusion distance; MHC, myosin heavy chain; sharing factor, mean capillary contacts per fiber/capillary-to-fiber ratio; μm, micrometers.

### Capillarization

Skeletal muscle capillaries were assessed as previously described (40, 41). Briefly, 6-μm sections were fixed in Carnoy’s solution for 5 min at RT, followed by a 30-min incubation (37°C) in 1% amylase with agitation, and then periodic acid-Schiff (PAS) staining as per manufacturer recommendation (Millipore Sigma; Burlington, MA; Cat. No.: 395B-1KT). Capillary density was calculated as the number of capillaries in a defined area (capillaries/mm^2^). Capillary-to-fiber ratio was calculated as the number of capillaries divided by the number of muscle fibers in the area (C:F). Local parameters of capillary contacts (CCs), sharing factor, CC per fiber area, maximal diffusion distance, and average diffusion distance were calculated. CC was determined by calculating the average number of fibers touching each capillary (total CC/total capillaries) (42) and similarly, sharing factor was determined by calculating the number of capillaries touching each muscle fiber (CC/C:F) (42). CC per fiber area (CC/FA, μm^2^·1,000) accounts for differences in fiber dimensions on potential diffusion (42). Maximal and average diffusion O_2_ distances from capillary to muscle fiber are based on the cumulative frequency of capillaries and the area of each fiber within a measured distance (43):

$$maximal\;diffusion\;distance\;=\;\left[0.415-\frac{0.477}{C:F}\right]\;\times \;\sqrt{FA}$$

$$average\;diffusion\;distance\;=\;\left[0.207+\frac{0.232}{C:F}\right]\;\times \;\sqrt{FA}$$

Maximal diffusion distance represents where 95% of the fiber area is served by a capillary, whereas average diffusion distance is the distance where 50% of the fiber area is served by a capillary (44). Collectively, these different methodological approaches provide a comprehensive view of muscle capillarization and diffusion that no one method capture alone. Capillarization is presented in Table 3.

### Picro Sirius Red Collagen Staining

Slides were stained with Picro Sirius Red Stain Kit as per manufacturer recommendations (Abcam; Cat. No.: ab150681) to reveal collagen content. Images were captured (×10 and ×40 magnification) with bright-field light. Staining intensities were analyzed from each baseline sample. Staining intensities were normalized to tissue area and the ratio of stained area to tissue area was calculated.

### Statistical Analysis

Because these analyses were derived from a previous study, allocation of samples to histology-based preservation was dependent on biopsy yield second to a required allocation of sample for analyses requiring homogenized tissue. Therefore, missing values are at random. To account for missing values, linear mixed model (LMM) regressions were developed using lmer function in R package lme4 (45) including fixed effect terms for time, fiber type, and random effect terms for participants to examine fiber-specific changes in response to exercise. Further examination into sexual dimorphisms used LMM regressions including fixed effect terms for sex, fiber type, and random effect terms for participants. LMM analysis was performed using R-Studio, version 1.2.5033, Release Orange Blossom (RStudio: Integrated Development for R., RStudio, Inc.; Boston, MA). Multiple comparisons were analyzed using lsmeans (46) function with Tukey adjustment. Spearman’s rank correlation coefficient was used to investigate relations between redox indices and participant characteristics, cardiometabolic fitness, and cellular features. Correlation analyses and descriptives were generated using Prism 9.4.1 software (GraphPad Software, Inc., La Jolla, CA). Data are expressed as means or percent differences ± 95% confidence intervals (95% CI). Significance was set at *P* < 0.05.

## RESULTS

### Fiber Type Redox Signatures

**Figure 1.**
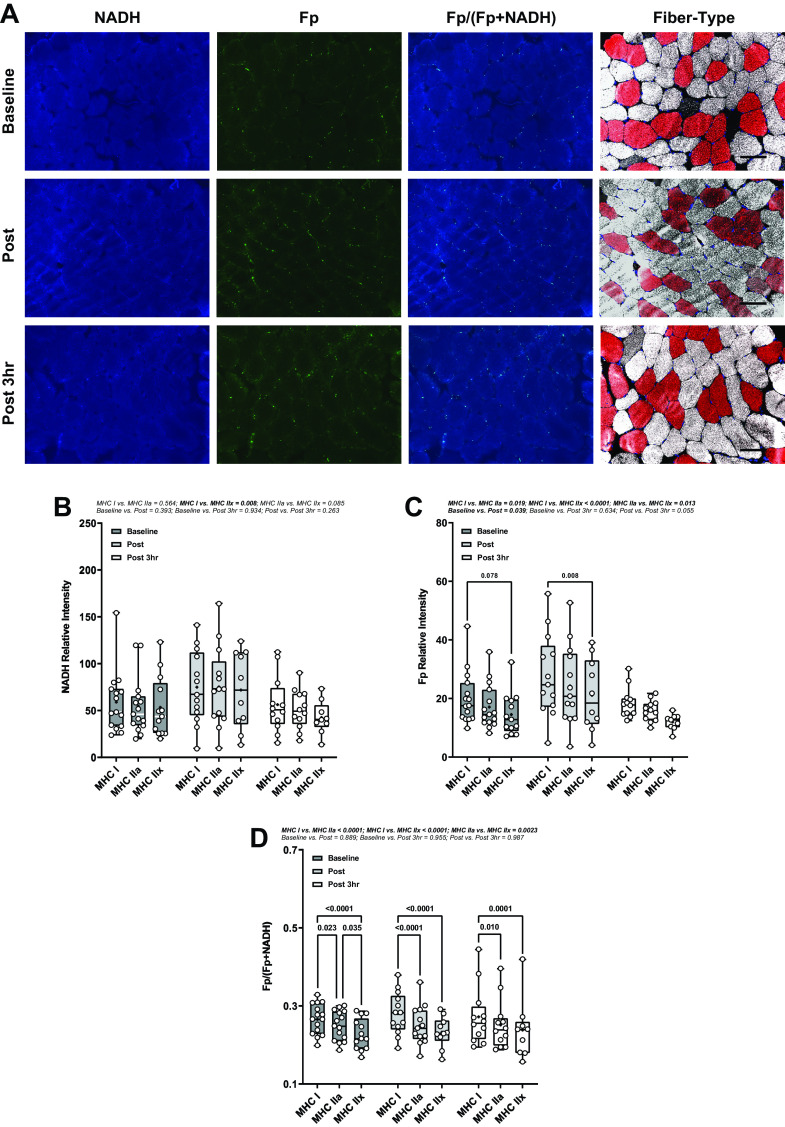
Fiber type-specific redox states in skeletal muscle. *A*: representative redox and fiber-typed images across Baseline (*n* = 15), Post (*n* = 13), and Post 3 h (*n* = 12) where *n* is number of subjects. Scale bar is 100 μm. *B–D*: redox relative intensities (A.U.) per fiber type. Data were analyzed using LMM. Box and whisker plots indicate mean (black dot) and 95% CI. Dots represent individual values of the redox indices, not all muscle sections contained MHC IIx. CI, confidence interval; LMM, linear mixed model; MHC, myosin heavy chain.

Skeletal muscle NADH and Fp signatures and the redox state are shown in Fig. 1*A*. At baseline, in a fasted state, there was no difference in NADH between fiber types (Fig. 1*B*), whereas Fp intensities trended to be elevated in MHC I compared with MHC IIx fibers by 60.7% (*P* = 0.078, 95% CI: 44.8, 76.5%). Redox ratios [Fp/(Fp + NADH)] were highest (more oxidized) in slow-twitch fibers favoring oxidative metabolism (e.g., MHC I), with a stepwise decrease in fast oxidative (e.g., MHC IIa) and fast glycolytic (e.g., MHC IIx). When comparing basal redox ratios among fiber types, the redox ratio of MHC I was 7.2% higher (*P* = 0.023, 95% CI: 5.2, 9.2%) than that of MHC IIa and the redox ratio of MHC IIa was 8.0% higher (*P* = 0.035, 95% CI: 6.8, 9.2%) than that of MHC IIx (Fig. 1*D*).

Further analyses were conducted after stratification by sex to identify sexual dimorphisms of redox indices (Supplemental Fig. S2). At baseline, there was no difference in NADH or Fp intensities between sexes within fiber types (Supplemental Fig. S2A). Baseline redox ratios of males were 23.1% higher than females in MHC I (*P* = 0.045) but not significantly elevated in MHC IIa (*P* = 0.126) or MHC IIx (*P* = 0.135).

### Redox Responses to Exercise

There was no effect of exercise or interaction with fiber type on NADH fluorescence intensity (Fig. 1*B*). Specifically, the NADH autofluorescent intensity remained lower in MHC IIx fibers than in MHC I (*P* = 0.0078) and MHC IIa fibers (*P* = 0.085) across all time points (Fig. 1*B*). However, compared with baseline, exercise increased muscle Fp fluorescence intensity immediately postexercise (*P* = 0.039) but not 3-h postexercise (*P* = 0.634; Fig. 1*C*). The fiber-specific pattern of Fp autofluorescence was lower in MHC IIx than in MHC IIa (*P* = 0.013) and MHC I fibers (*P* < 0.0001), whereas MHC IIa Fp autofluorescence was also lower than autofluorescence in MHC I fibers (*P* = 0.019) across all time points (Fig. 1*C*). Specifically, pairwise comparisons within the post time point of Fp revealed lower intensities in MHC IIx than in MHC I fibers by 19.0% (*P* = 0.008, 95% CI: 8.3, 29.7%; Fig. 1*C*). Despite the elevations in Fp following exercise, the redox ratio was not changed by exercise within or between fiber types (Fig. 1*D*).

Stratification by sex revealed a modest increase in muscle NADH of males compared with females (169.6 vs. 8.2%, *P* = 0.107). The increase in NADH caused a greater redox ratio shift toward an oxidized state in female muscle compared with male muscle after exercise (21.2 vs. −13.8%, *P* = 0.027, Supplemental Fig. S2B). Pairwise comparisons did not reveal differences in redox indices postexercise between sexes within fiber types. Furthermore, these sex-specific effects of NADH were more pronounced 3-h postexercise whereby muscle NADH of males rose by 24.4% but fell by 32.4% in females (*P* = 0.007, Supplemental Fig. S3B), largely driven by sex-specific differences in MHC I (*P* = 0.059) and MHC IIx (*P* = 0.022). No other effects of Fp or redox ratio were evident between sexes 3-h postexercise.

### Mitochondrial Markers

**Figure 2.**
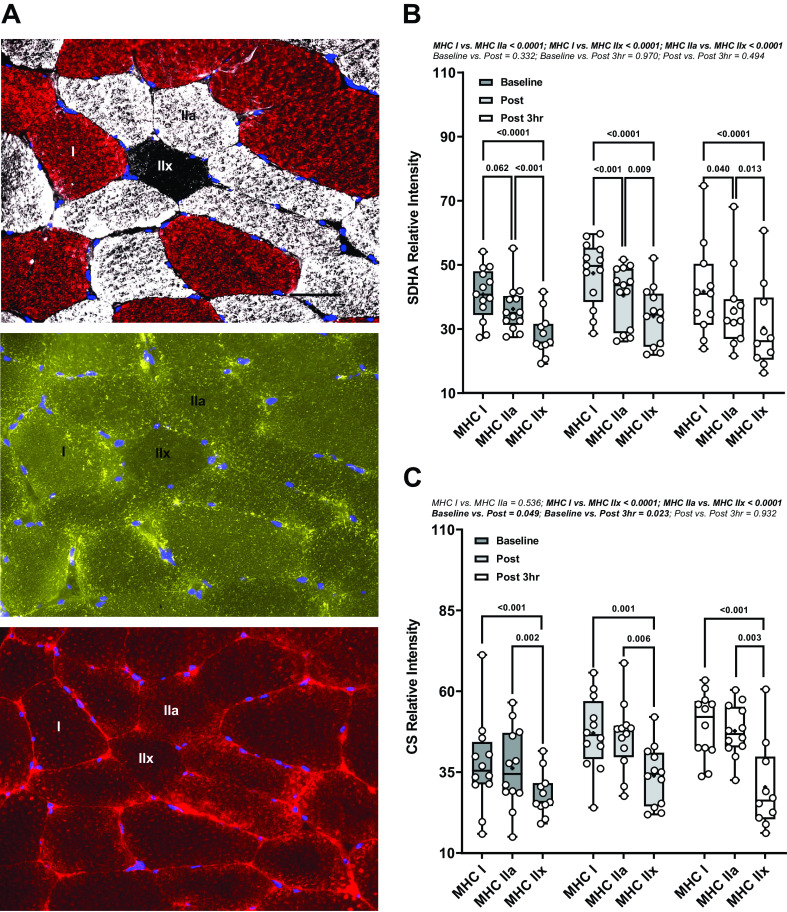
Fiber type-specific mitochondrial content in skeletal muscle. *A*: representative images of skeletal muscle fiber type (*top*), SDHA (*middle*), and CS (*bottom*). The scale bar is 50 μm. *B*: SDHA relative intensity (A.U.) per fiber type across Baseline (*n* = 13), Post (*n* = 13), and Post 3 h (*n* = 11) where *n* is number of subjects. *C*: CS relative intensity (A.U.) per fiber type across Baseline (*n* = 12), Post (*n* = 12), and Post 3 h (*n* = 12). Data were analyzed using LMM. Box and whisker plots indicate mean (black dot) and 95% CI. Dots represent individual values of the mitochondrial content indices, not all muscle sections contained MHC IIx. CI, confidence interval; CS, citrate synthase; LMM, linear mixed model; SDHA, succinate dehydrogenase subunit A.

Mitochondrial markers, SDHA and CS, followed classical fiber type phenotype at baseline whereby SDHA levels tended to be higher in MHC I than in MHC IIa fibers by 13.6% (*P* = 0.062, 95% CI: 8.6, 18.5%) and further elevated in MHC IIa compared with MHC IIx fibers by 29.7% (*P* < 0.001, 95% CI: 25.9, 33.6%; Fig. 2*B*). At baseline, CS levels were comparable between MHC I and MHC IIa fibers (*P* = 0.999). However, CS levels were lower in MHC IIx than in MHC I fibers by 48.9% (*P* < 0.001, 95% CI: 40.1, 57.8%) and MHC IIa by 45.1% (*P* = 0.002, 95% CI: 39.2, 50.9%; Fig. 2*C*). These fiber-specific distribution patterns were not affected by exercise and were thus maintained across all time points. However, main effects revealed CS was increased immediately postexercise (*P* = 0.049, 95% CI: 0.2, 12.8%) and 3-h postexercise in skeletal muscle (*P* = 0.022, 95% CI: 0.3, 21.0%; Fig. 2*C*).

No relations between basal NADH, Fp, or redox ratios with SDHA or CS within any fiber types (Supplemental Fig. S4) were observed. The post minus baseline change (Δ) in NADH exhibited a positive relationship to basal SDHA within MHC I (rho = 0.683, *P* = 0.050) and MHC IIa fibers (rho = 0.667, *P* = 0.059) but not in MHC IIx fibers (rho = 0.600, *P* = 0.350; Supplemental Fig. S5). We found no relations between ΔFp or Δredox ratio and basal SDHA or basal CS within any fiber type (Supplemental Fig. S5).

### Collagen

Picro Sirius red staining was used to identify collagen and to exclude the contribution of collagen autofluorescence to NADH intensity (see Supplemental Fig. S3). No significant relationships were found between NADH relative intensity and Picro Sirius red relative intensity (rho = −0434, *P* = 0.162) or the ratio of Picro Sirius red staining to total fiber area (rho = 0.210; *P* = 0.514) where collagen stained accounted for 0.75 ± 0.21% of the total fiber area within our cohort. We also did not observe any sexual dimorphism on collagen outcomes of Picro Sirius red relative intensity (*P* = 0.511) or the ratio of Picro Sirius red staining to total fiber area (*P* = 0.435).

### Correlations with the Exercise-Induced Changes in Redox Indices

Our next focus was to investigate the associations between exercise-induced changes in redox indices and anthropometrics, cardiorespiratory fitness, and cellular features (Supplemental Fig. S5). ΔRedox ratio was inversely correlated with absolute V̇o_2max_ (L/min) within MHC I (rho = −0.36, *P* = 0.013) and MHC IIa fibers (rho = −0.645, *P* = 0.037) but not associated within MHC IIx fibers (rho = −0.190, *P* = 0.665; Supplemental Fig. S5). Capillary density was positively correlated with Δredox ratio within MHC I (rho = 0.762, *P* = 0.037) and MHC IIa fibers (rho = 0.881, *P* = 0.007) and modestly in MHC IIx fibers (rho = 0.771, *P* = 0.103; Supplemental Fig. S5). Local parameters for diffusive capacity were also strongly related to Δredox ratio. We also found support for the link between absolute oxygen consumption and capillarization through an inverse relationship between absolute V̇o_2max_ and capillary density (rho = −0.518, *P* = 0.042). Together these correlations provide evidence for relations between fiber-specific redox states, capillarization, and absolute oxygen consumption.

## DISCUSSION

Measuring the autofluorescence properties of NADH and Fp within human skeletal muscle showed that NADH was not different between fiber types in the baseline fasted state but that slow oxidative type I fibers contained greater amounts of FAD than glycolytic type IIx fibers. In the baseline state, type I fibers also had a more oxidized redox state than type IIa and IIx fibers. The metabolic demand of exercise increased substrate oxidation as observed through immediate postexercise increases in Fp autofluorescence. Meanwhile, exercise-induced changes in NADH or redox ratios were not detected nor were there any fiber-specific differences in these changes. Correlational analyses revealed that the exercise-induced changes in the redox ratio in type I and IIa fibers were associated with absolute V̇o_2max_, suggesting that exercise-induced increases in redox ratio (toward a more oxidized state) may be greater in people with lower absolute oxygen consumption and body size. Exercise-induced changes in redox ratios were also positively associated with type I and IIa fiber capillarization, suggesting that local oxygen delivery also plays a role.

As the majority of NADH and Fp signals come from mitochondria (47, 48), our study helps to depict fiber-specific redox regulation in the mitochondria of human skeletal muscle. To our knowledge, this is the first study to characterize the fiber-specific properties of autofluorescent redox indices within human skeletal muscle and their response to acute exercise. Quantitative assessment of unfixed cross sections of muscle from C57Bl/6 mice has shown higher Fp in soleus (a predominantly type 1 fiber type muscle) than in gastrocnemius muscle (a mixed fiber type muscle) (49). Indeed, in a separate single fiber analysis of seven healthy men from Ren et al.’s study found that oxidative fibers contained the highest NADH content (50). However, the previous method used dissection of single fibers from the muscle bed and stained for myofibrillar ATPase while the rest of the fiber is analyzed for NADH by a bioluminescent technique, a tedious process. Our method departs from laborious methodologies using a feasible and timely autofluorescent methodology that supports findings of higher NADH levels in oxidative than in glycolytic fibers.

Our data elucidate redox responses to exercise within skeletal muscle and provide the associative factors influencing these responses. Our findings support the increased oxidation of substrate after moderate-to-high intensity (80% V̇o_2max_) exercise, as shown by elevations in Fp. Ren and colleagues also found NADH levels were reduced in oxidative fibers after low-intensity (40% V̇o_2max_) exercise, whereas reductions in NADH content within glycolytic fibers were uniquely evident after moderate (70% V̇o_2max_) and maximal (100% V̇o_2max_) exercise (50). These results are expected as fiber-type recruitment is dependent on metabolic demand. Surprisingly, we did not witness changes to redox ratios across time points within fiber types. Mitochondria under physiological stress can maintain the NAD/NADH ratio due to a large NAD(H) pool (51) and demonstrate a highly regulated inner membrane (52). It is possible changes in the redox ratio are maintained throughout one bout of exercise or the redox ratio rapidly returns to homeostasis upon the cessation of exercise within young healthy adults. However, an unexpected finding was the association between exercise-induced changes in redox state to absolute oxygen consumption. This observation may be attributed to our observed sexual dimorphisms whereby the redox state of female muscle was shifting to a more oxidized state, whereas male muscle shifts toward a more reduced state after exercise. Indeed, sex-specific differences in substrate utilization are often observed within humans (53). Females have greater reliance on fat with concomitantly lower CHO oxidation compared with males (54, 55). In previous findings, young men and women displaying similar aerobic exercise capacity, a higher mitochondrial density and increased capacity for fatty acid and lactate oxidation were found in female skeletal muscle supporting differences of mitochondrial properties between men and women (56). Moreover, data herein support previous reports (57) of higher capillary density in females than in males (*P* = 0.067, data not shown). Thus, opposing sex-specific redox responses to exercise in muscle may be underlined by mitochondrial parameters and local oxygen delivery. How these findings relate to sex-specific responses of exercise adaptation, disease-risk, and longevity will require more targeted study framework and deeper mechanistic insight.

Furthermore, we provide evidence for a relationship between exercise-induced changes in the redox state and muscle capillarization necessary for oxygen delivery. Adequate oxygen delivery is necessary for optimal oxidation of ETC substrate and redox regulation, adult cardiomyocyte models of ischemia-reperfusion display dramatic reduction in NAD/NADH within both cellular and mitochondrial compartments (58). Deficits in substrate utilization can lead to a decrease in NAD/NADH ratio and inhibit TCA cycle flux thus reducing NAD^+^ availability (18, 59). Reductions in free NAD^+^ reduce the activity of proteins requiring NAD^+^ as a cosubstrate, such as sirtuins, and hence affect cellular function (60, 61). On the other hand, compromised TCA cycle metabolism can decrease NADH production and increase the NAD^+^/NADH ratio, leading to oxidative stress (62), adding to the pathological role of redox dysregulation. The role of mitochondrial activity within the ETC and TCA cycle on skeletal muscle redox states would still need to be elucidated to confirm our results. Thus, our work suggests that the mitigation of microvascular and capillary dysfunction may optimize redox responses to exercise.

### Limitations

Despite the ease and utility of autofluorescent redox imaging, other endogenous fluorophores may contribute to measured emissions within skeletal muscle. Collagen and lipofuscins demonstrate detectable autofluorescent emissions found within wavelengths of 400–700 nm (63). Because collagen deposition mainly occurs within the extracellular matrix and intracellular lipofuscin aggregation is typically witnessed in old age or muscle dystrophy, it is unlikely these factors contributed to our analyses of intracellular muscle fibers of young healthy individuals. In addition, connective tissue was trimmed from muscle biopsy samples during processing, further minimizing the risk of a contribution to autofluorescent signal by collagens. NADPH also plays critical roles in the mitochondria and cytosol, not limited to the context of exercise, as a conduit for hydride transfer for several processes such as reduced glutathione regeneration in the mitochondria and NADPH oxidase-mediated superoxide production in the cytosol. This method cannot discern between NADH and NADPH autofluorescent emissions nor other conjugated proteins containing flavin groups. However, previous studies demonstrate that NADH fluorescence predominates over that of NADPH (64) and flavoproteins associated with pyruvate and α-ketoglutarate dehydrogenase predominate over other fluorescent flavoproteins of the respiratory chain within the utilized wavelengths (65). Amylase digestion-PAS staining also possess limitations in that fiber-specific capillary distributions cannot be discerned. Future studies should capitalize on the technical advantages of antibody-based multiplexing for capillary identification (e.g., anti-CD31) and fiber-type distributions on a single slide. It remains to be determined whether measurements made in the postprandial state would alter baseline and/or exercise-induced changes in skeletal muscle autofluorescence as relative oxidation of fat and CHO may be influenced by diet (66). It is important to highlight that all measures collected within the current study were made in an overnight fasted state. Moreover, the menstrual cycle may influence whole body substrate utilization during exercise, although much of the literature reports no effect (67–69). Despite the skeletal muscle autofluorescent redox response to exercise is unknown within humans, fitness status is likely to play a role after exercise under the predetermined exercise intensity (i.e., 80% V̇o_2max_) due to the known influence of fitness on substrate utilization (70). Although, all participants in the current study possessed similar cardiorespiratory fitness, were recreationally active, generally young, and were apparently free of chronic disease. Interindividual differences in lactate thresholds, glycogen and glucose mobilization, and peripheral perfusion may either influence or be delivered from differential mitochondrial respiratory capacity contributing to our results. Our knowledge can also be expanded regarding individual redox responses to varying aerobic exercise intensities.

### Conclusions

This study is the first to use autofluorescent imaging to describe differential redox states within human skeletal muscle fiber types at baseline and following aerobic exercise. In the fasted state, oxidative fibers had greater NADH, Fp, and oxidation-to-reduction redox ratios than glycolytic fibers and the exercised-induced change in redox states within slow oxidative and fast oxidative fibers were strongly related to local parameters of capillarization. These findings highlight an easy and efficacious technique for assessing skeletal muscle redox in humans. Future work should determine if these fiber-specific redox states are age-dependent and/or change postprandially, with training-induced changes in muscle capillarization, and/or are altered in the presence of metabolic disease.

## DATA AVAILABILITY

Data will be made available upon reasonable request.

## SUPPLEMENTAL DATA

10.6084/m9.figshare.23564385 Supplemental Figs. S1–S5 and Supplemental Table S1: https://doi.org/10.6084/m9.figshare.23564385.

## GRANTS

This work was supported in part by American Diabetes Association Junior Faculty Award 1-14-JF-32 and NIH R01 DK109948.

## DISCLAIMERS

Commercial funders had no control over the research design, data analysis, or publication outcomes of this work.

## DISCLOSURES

J.M.H. and T.P.J.S. have given invited talks at societal conferences and university/pharmaceutical symposia and meetings for which travel and accommodation were paid for by the organizers. J.M.H. and T.P.J.S. have also received research money from publicly funded national research councils and medical charities. T.P.J.S. has received research money from private companies, including Novo Nordisk Foundation, AstraZeneca, Amylin, AP Møller Foundation, and Augustinus Foundation. T.P.J.S. has consulted for Boost Treadmills and Gu Energy and owns a consulting business, Blazon Scientific, and an endurance coaching and education business, Veohtu. None of the other authors has any conflicts of interest, financial or otherwise, to disclose.

## AUTHOR CONTRIBUTIONS

E.R.M., R.K.P., and J.M.H. conceived and designed research; J.S., E.R.M., R.K.P., C.E.M., Z.L., K.N.L., and J.M.H. performed experiments; J.S., E.R.M., Z.L., K.N.L., J.T.M., and J.M.H. analyzed data; J.S., E.R.M., R.K.P., C.E.M., Z.L., K.N.L., J.T.M., T.P.J.S., and J.M.H. interpreted results of experiments; J.S., E.R.M., J.T.M., and J.M.H. prepared figures; J.S., E.R.M., J.T.M., T.P.J.S., and J.M.H. drafted manuscript; J.S., E.R.M., R.K.P., C.E.M., Z.L., K.N.L., J.T.M., T.P.J.S., and J.M.H. edited and revised manuscript; J.S., E.R.M., R.K.P., C.E.M., Z.L., K.N.L., J.T.M., T.P.J.S., and J.M.H. approved final version of manuscript.
